# Asymmetric trunk kinematics during gait is seen between concave side and convex side in adolescent idiopathic scoliosis

**DOI:** 10.1186/1748-7161-10-S1-O32

**Published:** 2015-01-19

**Authors:** Mitsuhiro Nishida, Kota Watanabe, Morio Matsumoto, Yoshiaki Toyama, Takeo Nagura

**Affiliations:** 1 Dept. of Orthopedic Surgery, Keio University, Tokyo, Japan; 2 Department of Advanced Therapy for Spine and Spinal Cord Disorders, School of Medicine, Keio University, Tokyo, Japan; 3 Dept. of Clinical Biomechanics, Keio University, Tokyo, Japan; 4 Japanese Red Cross Shizuoka Hospital, Shizuoka, Japan

## Objective

In Adolescent Idiopathic Scoliosis (AIS), clinical evaluations have been made using X-rays, CT and MR images. However, these images are insufficient in evaluating dynamic abnormality due to the spinal deformity. The purpose of this study was to clarify the characteristics of trunk kinematics of patients with AIS during a stance phase of gait.

## Patients and methods

Forty-one females with the pre-operative AIS participated in this study whose ages were 17.0±4.7 years old. Their spinal curve pattern according to Lenke's classification and curve severities by Cobb's angle were recorded. All the patients were asked to perform level walking on a 10m walk-way with their comfortable walking speed. Total of 28 reflective markers were placed on the both acromion, iliac crest, and upper/lower limbs, C7, Th10, and S1 representing the spinal shape. The marker positions were measured with eight camera opto-electronic motion capture system (OQUS, Qualysis, Sweden) and ground reaction force was measured by two force plates (AM6110, Bertec, USA). The trunk posture was defined by the markers of C7, S1 and the acromions. The angles relative to the pelvis were calculated as Euler-angle, were evaluated at the four time points on a stance phase; foot strike (FS), mid stance (MS), terminal extension (TE), and toe off (TO). Trunk symmetry was evaluated to compare the kinematics on sagittal, coronal and axial planes between concave side and convex side of the scoliosis by Wilcoxon signed-rank test (p<0.05).

## Results

Over all, axial trunk kinematics (degrees) of the concave side (FS (-6.7±4.4), MS (-6.2±3.9), TE (0.4±3.2) and TO (1.9±4.2)) were significantly different with those of the convex side (FS (-2.7±4.2), MS (-1.6±4.0), TE (4.8±3.7) and TO (6.3±3.9)). These axial balances of the trunk were shifted along to the concave side and supported the previous study. In particular, patients with Lenke type1 showed asymmetrical trunk kinematics in the axial balance while those of type5 showed no trunk asymmetry. Regarding the Cobb's angle, severities on Cobb's angle were not correlated with asymmetry of trunk posture during gait.

## Conclusions

In AIS patients, on a stance phase during gait, asymmetry in trunk angle was observed in the axial balance. The asymmetric trunk kinematics during gait was not affected by the severity of deformity but possibly affected by the position of major curve. Effect of other mechanical factors on trunk kinematics, such as flexibility of deformity, should be analyzed in the future study.

